# TgJosephin and TgRad23 are important for anti-IFN-γ virulence via deubiquitination of SPM1 in *Toxoplasma*

**DOI:** 10.1128/msphere.00137-26

**Published:** 2026-04-13

**Authors:** Emi Hashizaki, Yuta Tachibana, Junpei Fukumoto, Eizo Takashima, Hidetaka Kosako, Daisuke Okuzaki, Miwa Sasai, Masahiro Yamamoto

**Affiliations:** 1 Department of Immunoparasitology, Research Institute for Microbial Diseases, The University of Osaka 13013, Suita, Osaka, Japan; 2 Laboratory of Immunoparasitology, WPI Immunology Frontier Research Center, The University of Osaka 13013, Suita, Osaka, Japan; 3 Division of Malaria Research, Proteo-Science Center, Ehime University 12760, Matsuyama, Ehime, Japan; 4 Division of Cell Signaling, Institute for Advanced Medical Sciences, Tokushima University 215095, Tokushima, Japan; 5 Genome Information Research Center, Osaka University, Suita, Osaka, Japan; 6 The University of Osaka 13013, Suita, Osaka, Japan; 7 Department of Immunoparasitology, Center for Infectious Disease Education and Research, The University of Osaka 13013, Suita, Osaka, Japan; Virginia-Maryland College of Veterinary Medicine, Blacksburg, Virginia, USA

**Keywords:** Rad23, ataxin-3, TgJosephin, deubiquitinase, SPM1, IFN-γ, virulence, *Toxoplasma*, ubiquitin

## Abstract

*Toxoplasma gondii*, which can virtually infect all warm-blooded cells, secretes various virulence factors to evade interferon-gamma (IFN-γ)-dependent host immunity. While these secreted proteins are widely characterized, the molecular mechanisms important for virulence within the parasite are unclear. In this study, we aimed to investigate the roles of non-secretory proteins of *T. gondii* in immunosuppression. Deletion of deubiquitinase TgJosephin resulted in attenuated virulence in wild-type mice but not in mice lacking the interferon-gamma receptor (IFNγR). Moreover, TgJosephin expression was maintained by TgRad23, a protein involved in DNA repair and protein shuttling. Notably, TgJosephin depletion increased ubiquitination of subpellicular microtubule protein 1 (SPM1), a stabilizing component of the parasite microtubules, and mutating its ubiquitination sites restored virulence in the absence of TgJosephin. We propose TgJosephin as a novel virulence factor maintained by TgRad23 and a virulence pathway involving SPM1 metabolism.

**IMPORTANCE:**

*Toxoplasma gondii* is an obligate parasite whose infection can be detrimental when combined with pregnancy or immunodeficiency. Studies on *T. gondii* virulence have revealed various secretory proteins that inhibit the host interferon-gamma (IFN-γ) immune response. However, much of the broader virulence landscape remains unclear. To explore the unknown molecular pathways of *T. gondii* virulence in mice, we searched for immunosuppressive functions in genes encoding non-secretory proteins, associated with fundamental cellular processes of the virulent type I strain. Here, we found that TgJosephin, a highly conserved deubiquitinase, was important for virulence in wild-type mice but not mice lacking the IFN-γ receptor (IFNγR). In addition, TgJosephin expression was dependent on TgRad23, and loss of TgJosephin led to increased ubiquitination of a microtubule protein SPM1. Our results suggest a novel anti-IFN-γ pathway of *T. gondii* mediated by TgJosephin and SPM1 deubiquitination.

## INTRODUCTION

*Toxoplasma gondii* is a protozoan parasite that can virtually infect all warm-blooded animals and is estimated to have infected one third of the global population (1). Although most human infections are asymptomatic, infection during pregnancy or in immunocompromised individuals may result in severe toxoplasmosis (2). Understanding the molecular mechanisms of *T. gondii* virulence is important for treatment and prevention of toxoplasmosis (3). Upon invading a host cell, *T. gondii* forms a parasitophorous vacuole (PV) and proliferates within (1). Murine cells express various interferon-gamma (IFN-γ)-induced genes, which contribute to cell-autonomous parasite killing by immunity-related GTPase (IRG) activity on the PV membrane (4–6) and parasite growth inhibition by nitric oxide production and tryptophan degradation (7, 8). Highly virulent strains of *T. gondii* secrete various virulence factors from rhoptries and dense granules across the PV membrane to evade host immunity (9). While functions of these secreted proteins are widely studied, their regulators and non-secretory contributors to virulence are largely uninvestigated.

**Fig 1:**
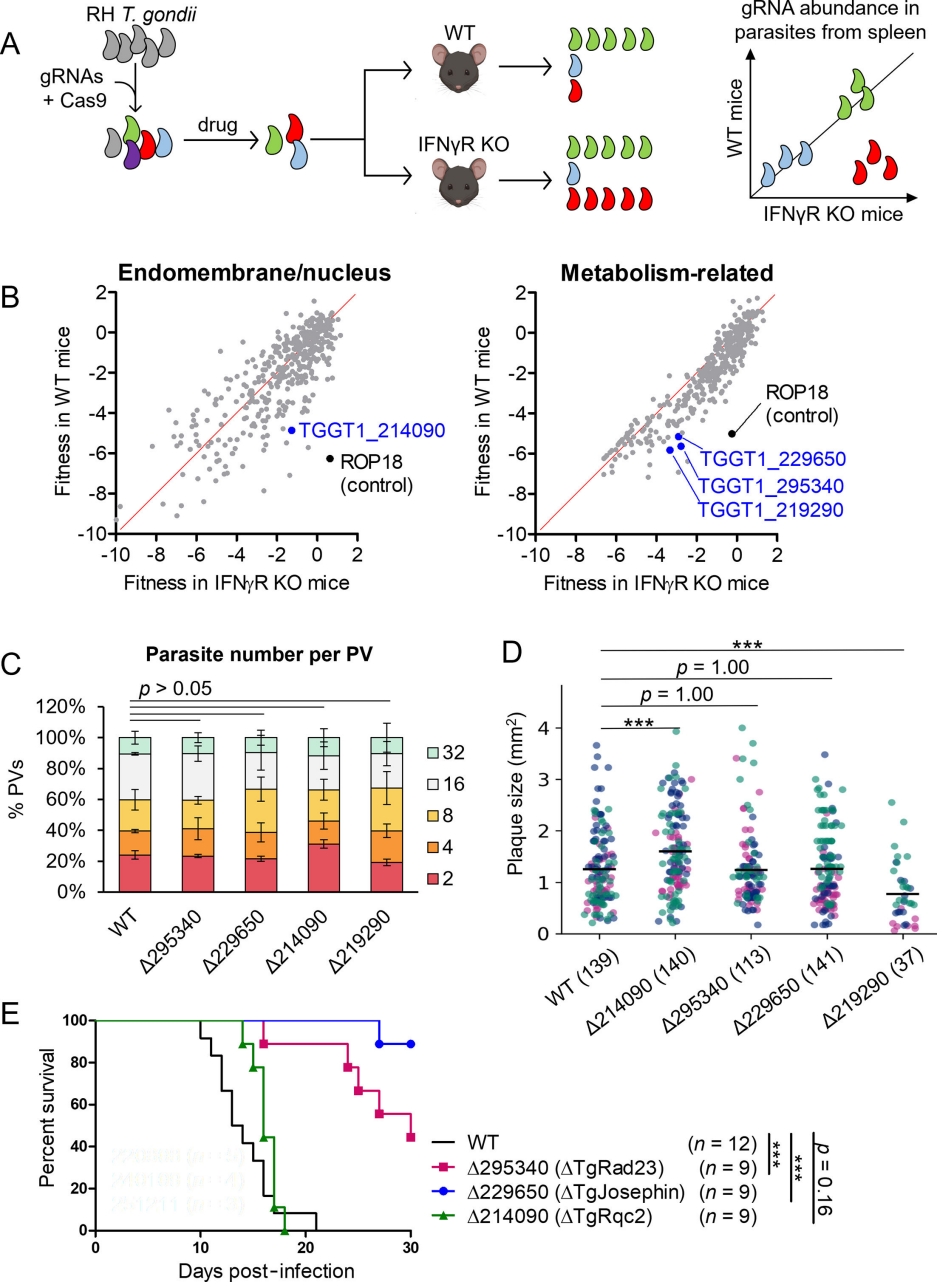
TgRad23 and TgJosephin deletion reduced virulence *in vivo*. (**A**) Schematic representation of the *in vivo* CRISPR screen made using https://BioRender.com. *T. gondii* mutants made with a gRNA library were selected in Vero cells with pyrimethamine and subcutaneously injected in footpads of mice. Mutations of parasites retrieved from the spleen were identified as belonging to the following categories: insignificant genes (green); genes important for parasite fitness *in vitro* (purple), *in vivo* (blue), and resistance to IFN-γ-dependent immunity (red). (**B**) Scatterplot showing *in vivo* fitness scores for each gene in WT and IFNγR-deficient mice in the indicated hyperLOPIT-determined sublibraries. Newly reported genes are marked in blue. ROP18 is marked in black as a control gene. (**C**) *In vitro* replication assay. 100 PVs per strain were counted for each experiment to compare parasite numbers per PV. *n* = 3. (**D**) Quantification of plaque sizes in five parasite strains in three experiments. Strain name (total plaque number in three experiments) and plaque size (mm^2^) are shown. The three experiments are distinguished by color. Data were compared with the Mann–Whitney test. ***P < 0.001. (**E**) Survival analysis of WT mice infected with the indicated parasites. ***P < 0.001.

In our previous study, an *in vivo* CRISPR screen was performed on the *T. gondii* genome using wild-type and IFN-γ-deficient mice, listing candidates for virulence-related genes in each sublibrary based on putative subcellular localization by hyperLOPIT (10, 11). Apart from known secretory proteins, highly ranked candidates included genes in the endomembrane-nucleus sublibrary and the metabolism-related sublibrary assigned to the cytosol, mitochondria, or apicoplast (Fig. 1A and B).

In this study, we investigated a signal peptidase (TGGT1_214090), recently named TgRqc2 (12), located in the non-chromatin region of the nucleus, and three putative metabolism-related proteins—TgRad23 (TGGT1_295340), TgJosephin (TGGT1_229650), and F-actin capping protein subunit beta TgCapZB (TGGT1_219290)—located in the parasite cytosol, as potential virulence factors. TgRad23 and TgJosephin are homologous to mammalian Rad23A/B and ataxin-3 (13–15) (Fig. S3A). Rad23A/B participates in nucleotide excision repair and protein shuttling to the proteasome (14, 15), while ataxin-3 is a deubiquitinase known for mutations that cause a neurodegenerative disorder called Machado–Joseph disease, or spinocerebellar ataxia type 3 (16, 17). The roles of their apicomplexan counterparts in virulence are relatively understudied. Our results suggest that *T. gondii* suppresses host IFN-γ-dependent immunity through regulation of SPM1 ubiquitination by TgRad23 and TgJosephin.

## RESULTS

### TgRad23 and TgJosephin deletion reduced virulence *in vivo*

Our CRISPR/Cas9 *in vivo* screening revealed TGGT1_214090, TGGT1_295340, TGGT1_229650, and TGGT1_219290, which encode a TgRqc2, TgRad23, TgJosephin, and a TgCapZB, respectively, as candidates for novel virulence genes against IFN-γ-mediated immunity. To investigate possible roles of TgRqc2, TgRad23, TgJosephin, and TgCapZB, we generated individual knockout type I parasites with CRISPR/Cas9 genome editing (Fig. S1A and B). First, parasite replication and plaque formation in unstimulated MEF monolayers were examined to assess whether the mutations affected parasite growth *in vitro*. All mutants showed normal replication in MEFs for 48 h (Fig. 1C). However, parasites lacking the TgCapZB (Δ219,290) produced smaller plaques in 8 days compared to the others, suggesting its vital role in *in vitro* growth (Fig. 1D; Fig. S5A). Since the aim of this study was to analyze their roles as virulence factors, TgCapZB was omitted from further experiments. In contrast, parasites lacking the TgRqc2, TgRad23, or TgJosephin could form normal-sized plaques (Fig. 1D; Fig. S5A). To assess whether these mutations affected parasite virulence in mice, wild-type mice were infected with 10^3^ wild-type or mutant tachyzoites via footpad injection. While mice infected with wild-type or TgRqc2-deficient strains all died within 17 days post-infection, mice infected with TgRad23- or TgJosephin-deficient strains showed significantly prolonged survival (Fig. 1E). Taken together, these results suggest that TgRad23 and TgJosephin are novel anti-IFN-γ virulence factors.

### TgJosephin and TgRad23 are important for suppression of IFNγ-dependent immunity

**Fig 2:**
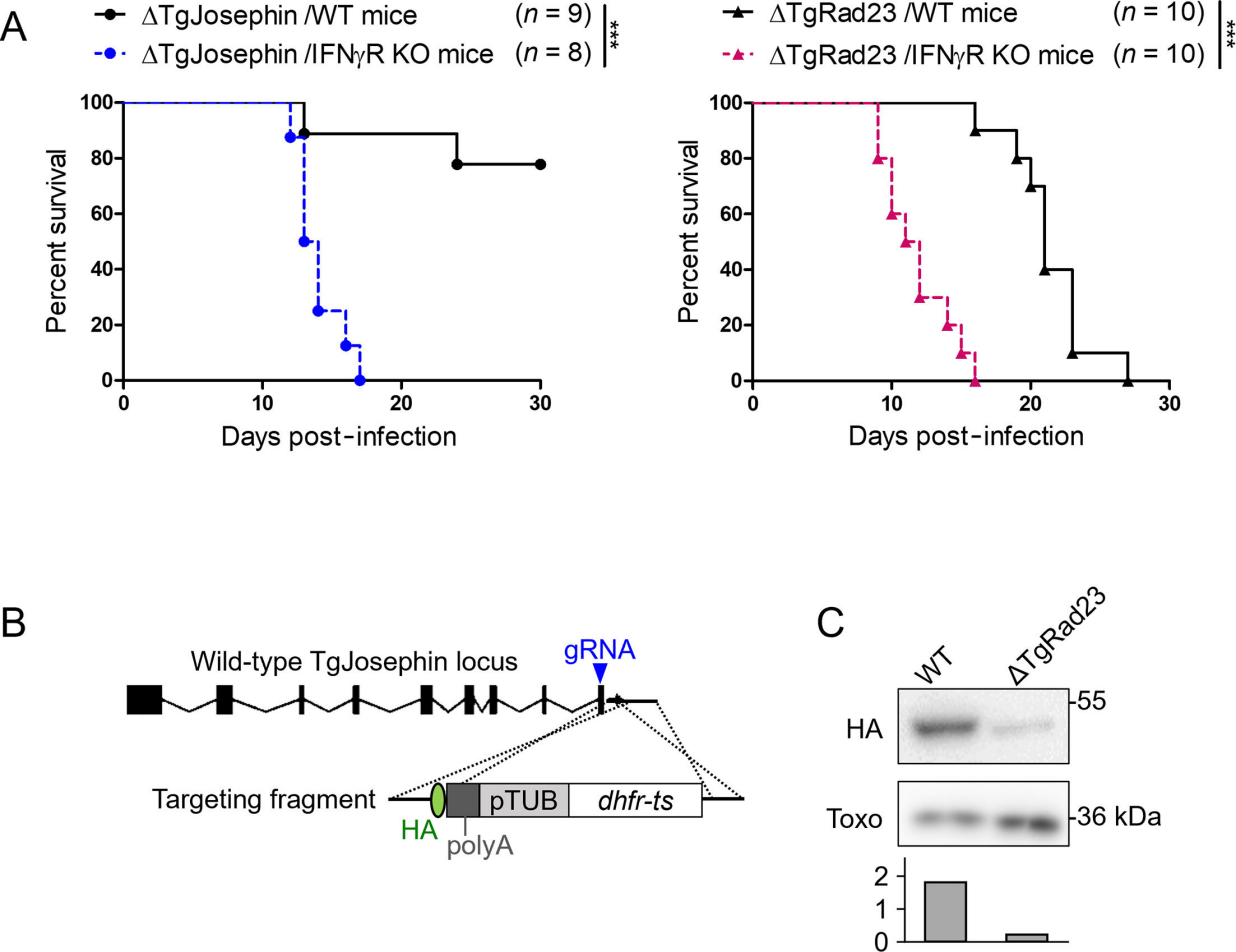
TgJosephin and TgRad23 are important for suppression of IFN-γ-dependent immunity. (**A**) Survival analyses of WT or IFNγR-deficient mice infected with TgRad23- or TgJosephin-deficient parasites. ***P < 0.001. (**B**) C-terminal tagging of TgJosephin in the endogenous TgJosephin gene region with an HA tag-containing gene cassette. (**C**) Western blot images of WT and TgRad23-deficient parasites both expressing an endogenous TgJosephin tagged with HA, stained with anti-HA and anti-*Toxoplasma* antibodies. Bar graph: HA/Toxo signal intensity ratio is shown for each lane.

To understand how TgJosephin and TgRad23 contribute to *T. gondii* virulence *in vivo*, we compared the survival of wild-type mice and mice lacking the IFN-γ receptor (IFNγR), both infected with parasites lacking TgJosephin or TgRad23. For both TgRad23 and TgJosephin mutants, all IFNγR knockout mice died within 17 days post-infection, while wild-type mice survived after 20 days (Fig. 2A). This suggests that TgJosephin and TgRad23 are important for specifically suppressing IFN-γ-mediated immunity.

Deletion of UBL-UBA shuttle proteins, including TgRad23, results in the accumulation of ubiquitinylated proteins (18). Ataxin-3, a homolog of TgJosephin, is known to interact with Rad23, and knockdown of Rad23 results in lower levels of ataxin-3 protein in mammalian cells and *Drosophila* (19), indicating that Rad23 maintains ataxin-3 protein expression. To investigate possible connections between the *T. gondii* counterparts TgRad23 and TgJosephin, a TgRad23-deficient strain was generated using type I parasites expressing an HA tag at the C-terminal region of the endogenous TgJosephin gene (Fig. 2B). HA-tagged TgJosephin detection was diminished in the TgRad23-deficient strain, suggesting that TgRad23 also maintains TgJosephin protein abundance (Fig. 2C).

### TgJosephin suppresses an IRG-independent immune pathway

**Fig 3:**
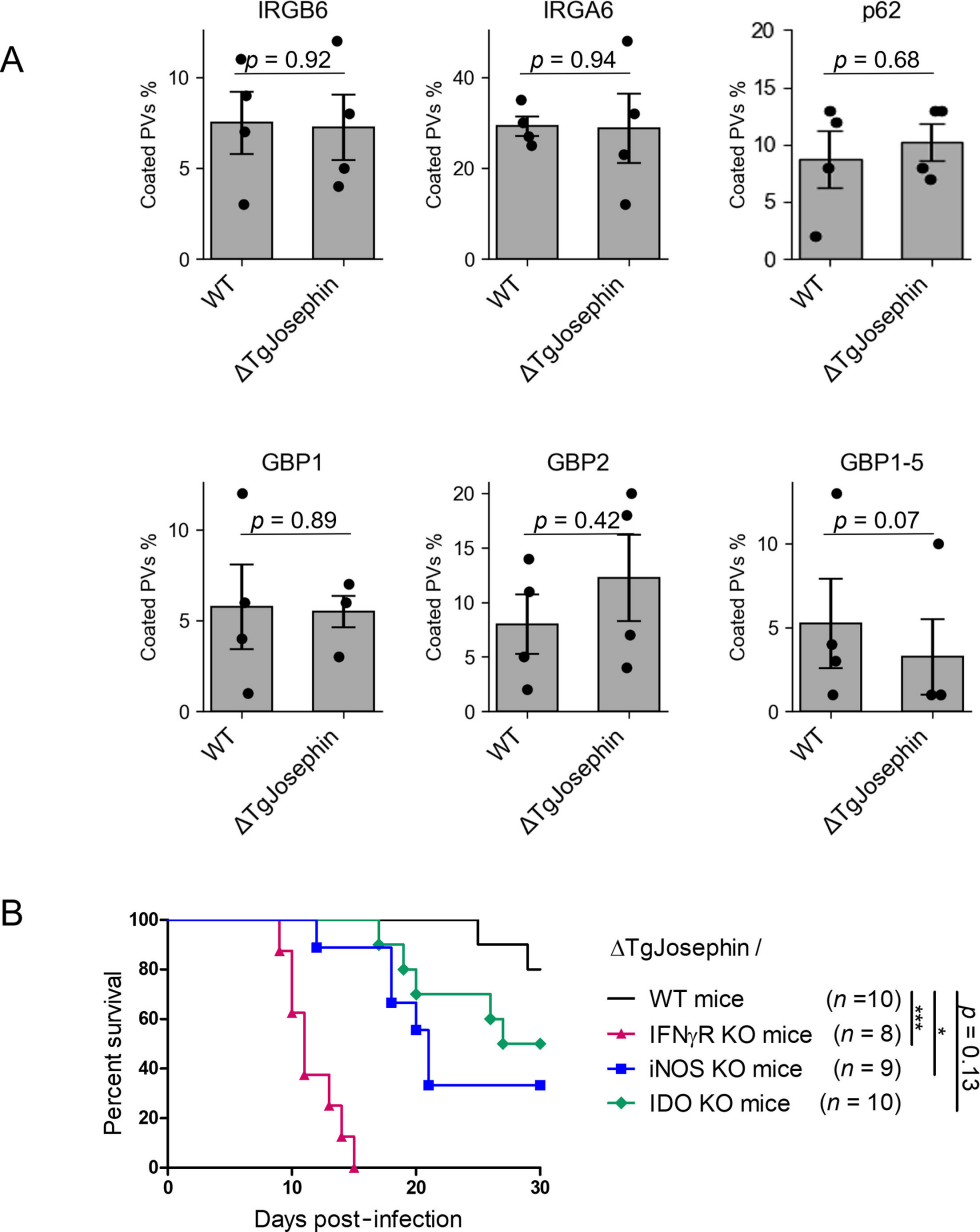
TgJosephin deletion did not increase loading of IFN-γ-induced proteins onto *T. gondii* PVs. (**A**) Graphs represent the percentage of Irgb6, Irga6, p62, GBP1, GBP2, and GBP1-5-coated PVs (marked with GRA5 or GRA17) at 2 h post-infection with WT or ΔTgJosephin parasites in MEFs treated with 10 ng/mL IFN-γ. 100 PVs were counted in each experiment. Mean ± SEM of four biological replicates is shown. (**B**) Survival analysis of WT mice or mice lacking IFNγR, iNOS, or IDO infected with TgJosephin-deficient parasites. *P < 0.05; ***P < 0.001.

To further investigate the mechanism by which TgJosephin inhibits IFN-γ-dependent immunity, we first tested *in vitro* markers of the cell autonomous immunity in wild-type and ΔTgJosephin parasites. TgJosephin-deficient parasites did not show increased loading of IFN-γ-inducible host factors IRGB6, IRGA6, p62, GBP1, GBP2, and GBP1-5 on PV membranes compared to wild-type parasites, suggesting that TgJosephin controls an immune suppression mechanism independent of IFN-inducible GTPase inhibition (Fig. 3A).

We then examined the virulence of ΔTgJosephin parasites in wild-type mice or mice lacking IFNγR, inducible nitric oxide synthase (iNOS), or indoleamine 2,3-dioxygenase (IDO). Thirty days post-infection, 80% of wild-type mice survived, while all mice lacking IFNγR died, and mice lacking iNOS and IDO were reduced to 33% and 50%, respectively (Fig. 3B). Compared to wild-type mice, IFNγR- and iNOS-deficient mice were significantly more susceptible to ΔTgJosephin parasites.

These results suggest that TgJosephin suppresses the growth inhibitory function mediated by iNOS, rather than PV coating and destruction by IFN-inducible GTPases.

### The deubiquitinase catalytic domain is important for TgJosephin-mediated virulence

**Fig 4:**
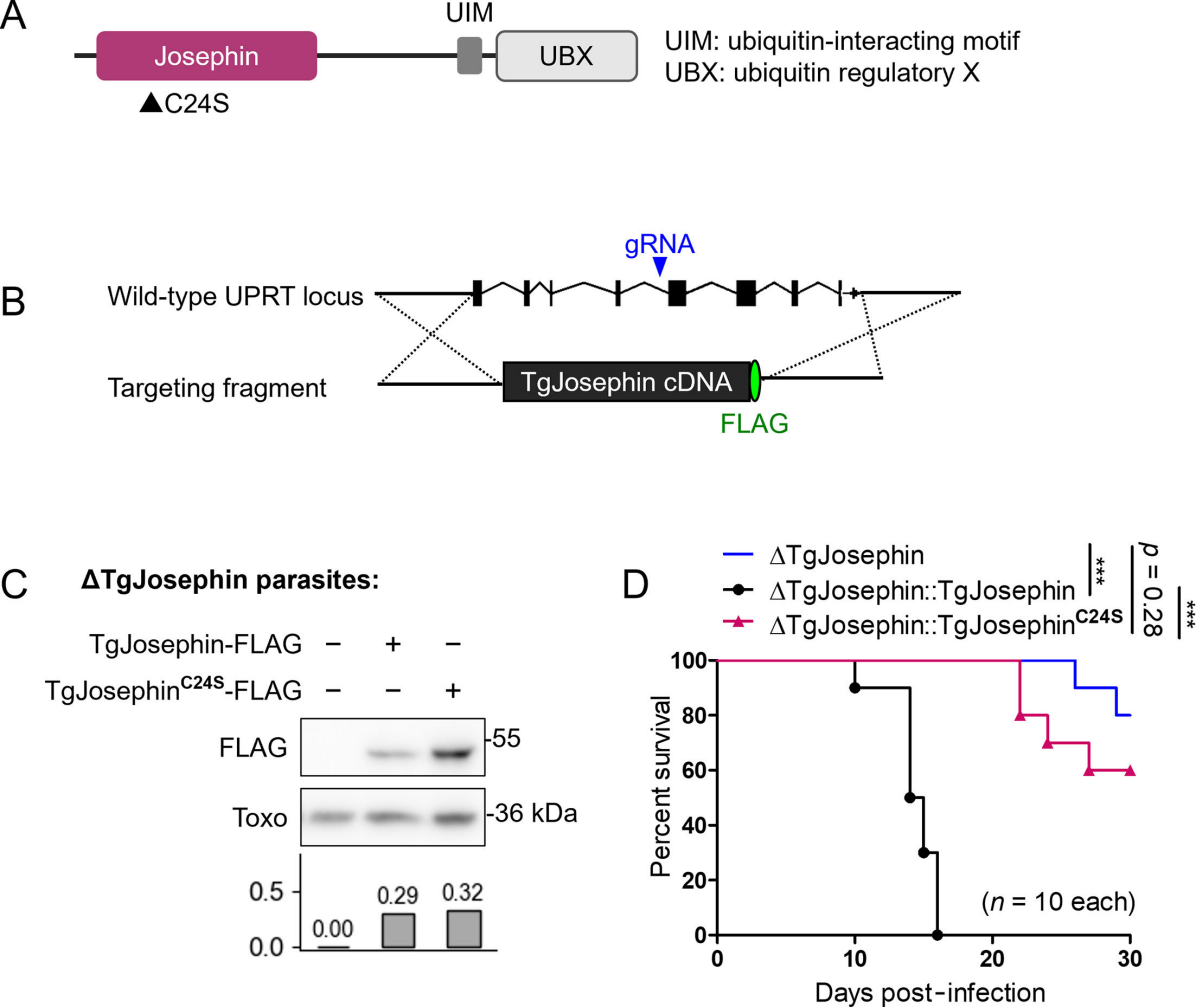
The deubiquitinase catalytic domain is important for TgJosephin-mediated virulence. (**A**) The Josephin domain, UIM, and UBX domain of TgJosephin with the C24S mutation site indicated below. (**B**) Complementation of TgJosephin in the UPRT (putative uracil phosphoribosyltransferase FUR1) locus of the TgJosephin-deficient strain. (**C**) Western blot analysis of FLAG-tagged TgJosephin in ΔTgJosephin parasites with an anti-*Toxoplasma* antibody detection as a positive control. Bar graph: FLAG/Toxo signal intensity ratio is shown for each lane. (**D**) Survival analysis of mice infected with TgJosephin-deficient parasites with or without complementation with TgJosephin and TgJosephin^C24S^. ***P < 0.001.

Previous studies in mammalian cells indicate that functions of Josephin family proteins include deubiquitination, aggresome formation, and docking of substrates to the proteasome (15, 20, 21). TgJosephin has the conserved catalytic domain (the Josephin domain), three ubiquin-interacting motifs, and a UBX domain, which is reportedly a protein-binding domain in other organisms (22) (Fig. 4A; Fig. S3B).

We aimed to confirm that the catalytic activity was critical to TgJosephin-mediated virulence. We searched for function-compromising mutation sites of the TgJosephin protein by aligning it with mammalian homologs using MEGA11 (23). Cysteine 24 of TgJosephin aligned with the active-site cysteine 14 of human ataxin-3 and cysteine 24 of human Josephin2, both of which are crucial for their catalytic enzyme activity (24–26).

To investigate whether mutation of this cysteine 24 to serine (C24S) affected *T. gondii* virulence *in vivo*, ΔTgJosephin parasites were complemented with a FLAG-tagged TgJosephin or TgJosephin^C24S^ cDNA in their putative uracil phosphoribosyltransferase FUR1 (UPRT) gene region and injected into the footpads of wild-type mice (Fig. 4A through D). Mice infected with parasites expressing wild-type TgJosephin all died within 16 days. On the other hand, mice infected with parasites expressing TgJosephin^C24S^ survived for 20–30 days (Fig. 4D). These results demonstrate that the deubiquitinase activity of TgJosephin is important for its role in immunosuppression.

### SPM1 ubiquitination was increased in TgJosephin-deficient parasites

**Fig 5:**
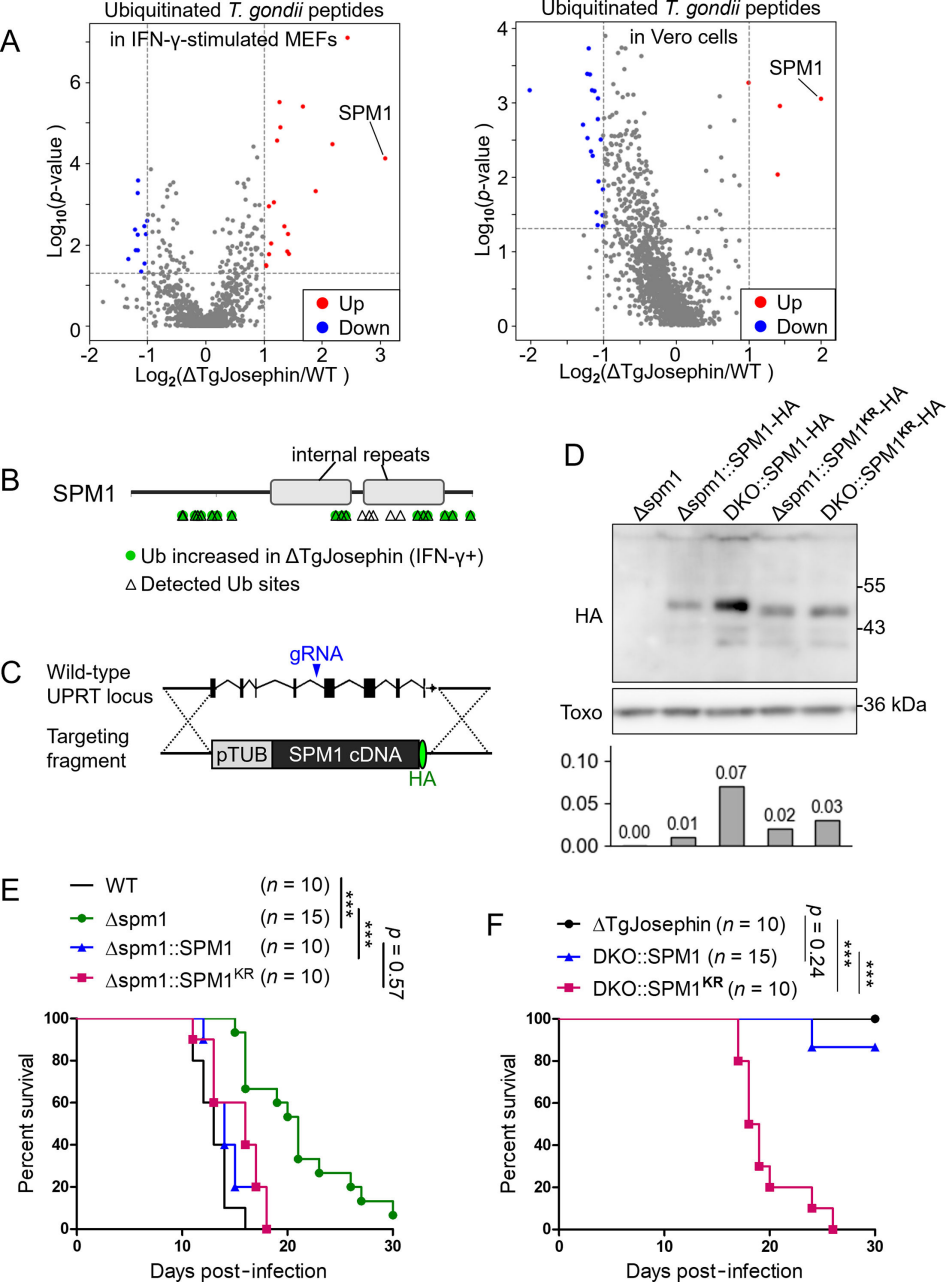
SPM1 ubiquitination was increased in TgJosephin-deficient parasites. (**A**) Volcano plots of differentially ubiquitinated peptides between WT and ΔTgJosephin parasites extracted from MEFs (left) and Vero cells (right). Abundance of each peptide was determined with ubiquitin-targeted mass spectrometry across four or five biological replicates each. Each dot represents a *T. gondii* peptide. (**B**) Schematic representation of the *T. gondii* SPM1 protein domain structure predicted by SMART. Arrowheads: lysine residues detected as ubiquitination sites by ubiquitin-targeted mass spectrometry. Sites with higher ubiquitination rates in ΔTgJosephin parasites compared to WT (*P* < 0.05, Student’s *t*-test) are marked in green. (**C**) Complementation of SPM1 in the UPRT locus of the Δspm1 strain. pTUB: alpha tubulin TUBA1 (TGME49_316400) promoter region. (**D**) Western blot analysis of HA-tagged SPM1 with anti-*Toxoplasma* antibody detection as a positive control. Parasite samples blotted with a mouse anti-HA antibody following immunoprecipitation with a rabbit anti-HA antibody. Bar graph: HA/Toxo signal intensity ratio is shown for each lane. (**E**) Survival analysis of WT mice infected with WT parasites, Δspm1 parasites, or Δspm1 parasites complemented with SPM1 or SPM1^KR^. (**F**) Survival analysis of WT mice infected with ΔTgJosephin parasites or DKO parasites complemented with either SPM1 or SPM1^KR^. ***P < 0.001. DKO: Δspm1ΔTgJosephin double knockout.

We searched for virulence factors among substrates of TgJosephin by comparing ubiquitinated peptide abundances in wild-type and ΔTgJosephin tachyzoites isolated from Vero or IFN-γ-stimulated MEF cells using ubiquitin-targeted mass spectrometry. No significant changes in ubiquitination of known virulence factors, such as GRA proteins and ROP proteins, were found between wild-type and ΔTgJosephin strains. Meanwhile, the highest increase rate of ubiquitination by TgJosephin deletion was found in microtubule-associated protein SPM1 (Fig. 5A and B; Table S2). On the other hand, a quantification of *T. gondii* proteins by data-independent acquisition (DIA) showed no difference in SPM1 protein abundance between WT and TgJosephin-deficient strains (Fig. S5B and Table S3), suggesting that TgJosephin deletion affected the ubiquitination, but not degradation or expression, of SPM1.

To investigate the significance of SPM1 ubiquitination in parasite virulence, we generated SPM1-knockout (Δspm1) and SPM1-TgJosephin double knockout (DKO) parasites and complemented them with tagged SPM1 or the mutant SPM1^KR^, in which all the detected ubiquitin target lysine residues were mutated to arginine without affecting protein folding (Fig. 5B and C; Fig. S4B, D, and E). Expression of SPM1 and SPM1^KR^ was confirmed by western blotting and RT-qPCR (Fig. 5D; Fig. S4C).

SPM1 is a component of the subpellicular microtubules of apicomplexans that is important for *T. gondii* viability and resistance of microtubules to detergent treatment *in vitro* (27, 28). There are currently no reports on the role of *T. gondii* SPM1 in IFN-γ-stimulated cells or immunosuppression *in vivo*. Interestingly, deletion of SPM1 resulted in a mild virulence defect in wild-type mice, for which SPM1 and the mutant SPM1^KR^ could equally complement (Fig. 5E; Fig. S4A).

Next, to address whether increased ubiquitination of SPM1 affected immunosuppression in TgJosephin-deficient parasites, wild-type mice were infected with ΔTgJosephin or Δspm1ΔTgJosephin (DKO) strains complemented with SPM1 or SPM1^KR^. Notably, all mice infected with parasites lacking TgJosephin and expressing normal SPM1 survived for over 30 days after infection, while mice infected with the SPM1^KR^-expressing strain died within 20 days (Fig. 5F). These data suggest that SPM1 ubiquitination is generally harmless to the parasite but can impede its virulence in TgJosephin-deficient conditions.

## DISCUSSION

We have found TgJosephin as a novel virulence factor of *T. gondii*. We aimed to discover unknown pathways and molecules involved in immunosuppression by screening gene knockout strains for parasite proliferation in mice and survival of infected mice. We selected candidates predicted to be proteins resident in the parasite nucleus or cytosol and unlikely to bind host factors as conventional virulence factors do. Our study suggests that TgJosephin is important for adequate virulence in wild-type mice but not in IFNγR-deficient mice, and TgRad23 is important for abundant TgJosephin expression. The relatively mild virulence impairment of ΔTgRad23 parasites may be attributed to inadequate expression of TgJosephin, whose total absence leads to a sharper loss of virulence. As TgJosephin deletion did not increase host IRG and GBP recruitment on PVs, TgJosephin likely suppresses a pathway of the IFN-γ-mediated response independent of IFN-inducible GTPases. Furthermore, deubiquitination activity of TgJosephin was suggested as the key to its role in virulence, and TgJosephin deficiency causes increased ubiquitination of SPM1, an impediment to *T. gondii* virulence in mice.

We propose that TgJosephin, maintained by TgRad23, inhibits a pathway in the IFN-γ response, apart from PV coating by IFN-inducible GTPases, through deubiquitination and clearance of abnormal SPM1. The mechanism by which SPM1 deubiquitination by TgJosephin suppresses host IFN-γ-dependent immunity remains unclear.

While deletion of TgJosephin may have a subtle effect on parasite growth in IFN-γ-stimulated murine bone-marrow macrophages (BMDMs) (Fig. S5C), it did not affect invasion, egress, or microtubule morphology *in vitro* (Fig. S5D through F). Still, we cannot say that over-ubiquitination of SPM1 does not alter the formation or function of subpellicular microtubules *in vivo* (29). Interestingly, SPM1 appears to have an anti-IFN-γ function, as its deletion reduced virulence in wild-type but not IFNγR-deficient mice (Fig. S4A). Our results suggest that wild-type SPM1 promotes immunosuppression in the presence of TgJosephin. Without TgJosephin, SPM1 undergoes uncontrolled ubiquitination, becoming dysfunctional or even inhibitory to anti-IFNγ virulence *in vivo*, whereas replacement of its Lys residues bypasses this effect. We propose that TgJosephin preserves SPM1 as a pro-virulence factor by restraining its ubiquitination.

As both inhibitory and promotive traits of Josephin family deubiquitinases in protein degradation have been discussed (30–32), the lack of deubiquitination of SPM1 by TgJosephin could lead to scarce SPM1 expression due to increased degradation or an abnormal pool of ubiquitinated SPM1. Given that the SPM1-HA signal in DKO::SPM1 parasites was no weaker than in Δspm1::SPM1, Δspm1::SPM1^KR^, or DKO::SPM1^KR^ (Fig. 5E), and that SPM1 protein abundance did not significantly change with TgJosephin deletion (Fig. S4B), wild-type SPM1 expression while lacking TgJosephin expression may not result in over-degradation of SPM1, but rather in a relative increase in ubiquitinated SPM1.

Ubiquitination, protein expression, and mRNA expression of known virulence proteins were not prominently affected by TgJosephin deletion (Tables S2 to S4). Still, we cannot exclude the possibility that multiple substrates of TgJosephin are involved. In ΔTgJosephin parasites compared to wild-type parasites, while SPM1 had an outstanding number of increased ubiquitination sites in both Vero cells and stimulated MEFs, SPM2 (TGGT1_286590), the hypothetical protein (TGGT1_220510), and the HMG (high mobility group) box domain-containing protein (TGGT1_263720) also exhibited increased ubiquitination at multiple lysine residues (Table S2). Since SPM2 is also localized in subpellicular microtubules (19), its overubiquitination in TgJosephin deficiency may have similar consequences on parasite virulence as SPM1. The TGGT1_263720 is homologous to HMGB1, a DNA binder that functions as a transcriptional regulator in the nucleus (33, 34). A bulk RNA sequencing experiment showed that some genes were upregulated or downregulated in the ΔTgJosephin strain compared to the wild-type (Table S4), all of which seemed irrelevant to immunosuppression, while the possibility of unknown virulence mechanisms involving these genes cannot be excluded.

Given that TgJosephin deletion did not affect the patterns of PV coating by Irgb6, Irga6, p62, or GBP1/2/1-5, ΔTgJosephin parasites in wild-type mice have likely succumbed to IFN-γ-driven growth inhibition instead of cell-autonomous cytotoxicity. The difference between mice lacking IFNγR and iNOS or IDO in Fig. 3 suggests that TgJosephin represses multiple pathways in the IFN-γ-induced response, including nitric oxide production by iNOS and possibly tryptophan starvation by IDO. Although IDO (IDO1 and IDO2) deficiency did not significantly increase susceptibility to ΔTgJosephin parasites, simultaneous deletion of iNOS and IDO may affect host survival to a degree comparable to that observed with deletion of IFNγR.

IFN-γ-dependent nitric oxide increase has been reported *in vitro* (35, 36), and a recent study showed evidence for iNOS-dependent nitration around *T. gondii* in infected cells (37). Sodium nitroferricyanide(III) dihydrate (SNP) has been used to mimic cellular nitric oxide production (38–40). When tachyzoites were exposed to 0–2 mM SNP prior to MEF infection to test their sensitivity to nitric oxide, we found no significant difference between wild-type and ΔTgJosephin parasites (Fig. S5G). This could suggest that interference of TgJosephin in the iNOS-mediated pathway precedes nitric oxide metabolism in the parasite, although we are yet to establish a method to study the importance of TgJosephin in virulence-related phenotypes *in vitro*.

Our findings suggest the importance of the TgRad23−TgJosephin−SPM1 axis in suppression of IFN-γ-driven immunity. It is worth noting that, unlike virulence factors of rhoptries and dense granules, TgRad23 and TgJosephin have homologs in mammals. These atypical virulence factors highlight the need to extend our study of *T. gondii* immunosuppression to genes conserved beyond apicomplexans and genes with basic functions.

## MATERIALS AND METHODS

### Cell culture and mice

C57BL/6NCrSlc (C57BL/6N) mice were purchased from SLC. Mice lacking IFNγR were described previously (41). For CRISPR/Cas9 targeting of iNOS and IDO, T7 promoter was added to the gRNA templates (iNOS_gRNA1 and iNOS_gRNA2 for iNOS, IDO1_gRNA and IDO2_gRNA for IDO) using KOD FX NEO. These gRNA and Cas9 mRNA were purified and used to generate iNOS- or IDO-deficient mice, as previously done for GNAQ-deficient mice (42).

All animal experiments were conducted with the approval of the Animal Research Committee of the Research Institute for Microbial Diseases in Osaka University.

*T. gondii* and mammalian cells were cultured at 37°C in 5% CO_2_. *T. gondii* strains were maintained in Vero cells by bi-weekly passage in RPMI (Nacalai Tesque) supplemented with 2% heat-inactivated fetal calf serum (FCS; JRH Bioscience), 100 U/mL penicillin, and 0.1 mg/mL streptomycin (Nacalai Tesque). All parasites used were tachyzoites of RHΔhxgprtΔku80 and its derivatives.

Mouse embryonic fibroblasts (MEFs) were grown in DMEM (Nacalai Tesque) supplemented with 10% FCS, 100 U/mL penicillin, and 0.1 mg/mL streptomycin (Nacalai Tesque).

Murine macrophages were induced from the marrow of the pelvis, femur, and tibia. Bone marrow was collected and washed with phosphate buffered saline (PBS) before and after ammonium-chloride-potassium (ACK) lysing buffer suspension and cultured for 6 days in 70% RPMI 1640-based medium (containing 10% FCS, 100 U/mL penicillin, and 0.1 mg/mL streptomycin) + 30% L-cell supernatant. For iNOS detection, cells were stimulated with 1 μg/mL lipopolysaccharide (LPS) overnight on the sixth day (Fig. S2B).

### Reagents

Goat polyclonal antibody against Irgb6 (TGTP; sc-11079) was purchased from Santa Cruz Biotechnology, Inc. Rabbit polyclonal anti-GBP2 and mouse monoclonal anti-p62 (H00008878-M01J) antibodies were obtained from Proteintech and Abnova, respectively. Recombinant mouse IFN-γ was purchased from PeproTech. Other reagents are listed in Table S1B.

### Plasmid construction for generation of KO *T. gondii* strains

For construction of the CRISPR/Cas9 plasmids targeting TgRqc2 (TGGT1_219290), TgRad23 (TGGT1_295340), TgJosephin (TGGT1_229650), TgCapZB (TGGT1_219290), and SPM1 (TGGT1_263520), two oligonucleotide primers (TgRqc2_gRNA1_F and TgRqc2_gRNA1_R, TgRqc2_gRNA2_F and TgRqc2_gRNA2_R for TgRqc2, TgRad23_gRNA1_F and TgRad23_gRNA1_R, TgRad23_gRNA2_F and TgRad23_gRNA2_R for TgRad23, TgJosephin_gRNA1_F and TgJosephin_gRNA1_R, TgJosephin_gRNA2_F and TgJosephin_gRNA2_R for TgJosephin, TgCapZB_gRNA1_F and TgCapZB_gRNA1_R, TgCapZB_gRNA2_F and TgCapZB_gRNA2_R for TgCapZB, SPM1_gRNA1_F and SPM1_gRNA1_R, SPM1_gRNA2_F and SPM1_gRNA2_R for SPM1) containing gRNA sequences were annealed and cloned into the BsaI cleavage site of the Cas9-pU6-Universal vector.

Targeting fragments containing a floxed HXGPRT cassette between two 60 bp genomic regions outside the PAM sequences were amplified using the following primers: TgRqc2_targeting_F and TgRqc2_targeting_R; TgRad23_targeting_F and TgRad23_targeting_R; TgJosephin_targeting_F and TgJosephin_targeting_R; TgCapZB_targeting_F and TgCapZB_targeting_R; and SPM1_targeting_F and SPM1_targeting_R.

Primer sequences are listed in Table S1A. Primers and gRNA were designed using *T. gondii* GTI genome sequences from ToxoDB.org (43).

### Gene-targeted CRISPR/Cas9 genome editing of *T. gondii*

In all transfection procedures of *T. gondii*, tachyzoites were filtered and resuspended in Cytomix (10 mM KPO_4_, 120 mM KCl, 0.15 mM CaCl_2_, 5 mM MgCl_2_, 25 mM HEPES, 2 mM EDTA) supplemented with 2 mM ATP (Wako) and 5 mM Glutathione (Nacalai Tesque). Cells were electroporated with GENE PULSER II (Bio-Rad Laboratories). Mutant parasites were selected with drugs in Vero cell culture or by sorting and then separated into clones by limiting dilution in MEF monolayers in 96-well plates.

### Deletion of *T. gondii* genes by CRISPR/Cas9 genome editing

The RHΔhxgprtΔku80 strain expressing firefly luciferase was transfected with 50 µg each of gRNA1 and gRNA2 CRISPR plasmids, along with the PCR-amplified targeting fragment for each gene. Mutants were passaged in 25 µg/mL mycophenolic acid (Sigma) and 50 µg/mL xanthine (Wako). Mutant clones were obtained by limiting dilution and screened by quantitative RT-PCR, using primers listed in Table S1A.

For the Δspm1ΔTgJosephin double knockout, the HXGPRT cDNA was deleted in Δspm1 parasites using a Cre-YFP vector. Δspm1Δhxgprt cells were selected by 80 μg/mL 6-thioxanthine (Santa Cruz) and used for TgJosephin gene deletion.

Cells expressing the endogenously tagged TgJosephin-HA were used for TgRad23 deletion (Fig. 2B).

### Protein tagging in the endogenous TgJosephin gene region

An HA tag sequence conjoined with the polyA tail and promoter regions of TGME49_316400 alpha tubulin TUBA1 and DHFR-TS cDNA was inserted in the C-terminal region of the endogenous TgJosephin gene locus of ΔTgJosephin parasites.

### Complementation of TgJosephin and SPM1 in TgJosephin- and SPM1-deficient *T. gondii*

*T. gondii* cDNA sequences were obtained from ToxoDB.org (43). To complement TgJosephin-deficient parasites, full-length TgJosephin cDNA, with or without the C24S mutation, was amplified by PCR using the RHΔhxgprtΔku80 cDNA as template and inserted into the pBlunt vector.

The cDNA fragments were cleaved and inserted into the pBlueScript plasmid vector containing the 3′-terminal and 5′-terminal UTRs of the putative uracil phosphoribosyltransferase FUR1 (UPRT) gene.

To complement SPM1-deficient parasites, full-length SPM1 cDNA was amplified by PCR using the RH *T. gondii* cDNA as template and inserted into the pBlunt vector. A synthetic mutant SPM1^KR^ cDNA was ordered from FASMAC, in which 20 lysine residues detected as ubiquitination sites by mass spectrometry (Table S2) were substituted with arginine (Fig. S4B).

The SPM1 and SPM1^KR^ cDNA were cleaved and inserted into the pBlueScript plasmid vector containing the promoter and polyA tail regions of TGME49_316400 alpha tubulin TUBA1 and the 3′-terminal and 5′-terminal UTRs of the UPRT gene.

TgJosephin- and SPM1-deficient *T. gondii* parasites were transfected with plasmids containing UPRT_gRNA and their respective cDNA fragments (50 µg each).

Transfected parasites were selected with 10 µg/mL fluorodeoxyuridine (FUDR) (Wako).

### Gene expression measurement by quantitative PCR

Tachyzoites were pelleted and used for total RNA extraction using QIAGEN RNeasy Mini Kit. RNA samples were incubated at 70°C for 2–3 min and used to synthesize cDNA with Thermo Scientific Verso cDNA Synthesis Kit. RT-PCR was performed using the synthesized cDNA and Promega GoTaq qPCR Master Mix on a BIO-RAD CFX Connect Real-Time System. Expression data were taken by the ΔΔCt method relative to TgACT1 (TGGT1_209030) expression levels.

### Western blotting

Cells were lysed in lysis buffer containing 1% protease inhibitor cocktail (Nacalai Tesque). Cell lysates or immunoprecipitated samples were separated by SDS-PAGE, transferred to Immobilon-P PVDF membranes (Millipore), and blotted with the indicated antibodies in 5% skim milk (Difco Laboratories). Membranes were immersed in Luminata Forte Western HRP substrate (Millipore) and used for signal detection with an ImageQuant LAS 4000 system (GE Healthcare).

The *Toxoplasma gondii* (1.B.516): sc-73210 antibody by Santa Cruz Biotechnology, Inc. (referred to as “Toxo” in figures) was used as a loading control for *T. gondii* samples.

### Immunoprecipitation

Cells were lysed in lysis buffer (1% NP-40, 150 mM NaCl, 20 mM Tris-HCl, pH 7.5) containing 1% protease inhibitor cocktail (Nacalai Tesque). Cell lysates were mixed with Protein G Sepharose 4 Fast Flow (Cytiva) and rotated for 30 min. Lysates were centrifuged, and supernatants were mixed with an antibody (1 μg) and rotated overnight. Protein G Sepharose 4 Fast Flow was added to the samples and rotated for 1 h, followed by washing with lysis buffer.

### Assessment of *T. gondii* virulence in mice

Mice were infected with 1.0 × 10^3^ *T. gondii* tachyzoites in 50 μL PBS per mouse by footpad injection. Survival was monitored for 30 days post-infection. Kaplan–Meier survival curves are shown; statistical significance was determined using the log-rank (Mantel–Cox) test. Each data set consists of two independent experiments (replicates).

### Plaque assay

Two hundred tachyzoites were added onto a primary MEF monolayer in a 6-well plate and cultured for 8 days. Cells were washed with PBS, fixed for 30 min with 4% formaldehyde, and then stained with 1% crystal violet. Photos were taken at 4× magnification using an optical microscope (Olympus CKX53).

### Immunofluorescence assay

Cells on coated coverslips were fixed with 4% formaldehyde in PBS for 10 min, permeabilized with 0.002% digitonin in PBS for 10 min, and blocked with 8% FCS in PBS for 10 min. Cells were co-stained with the indicated primary antibodies for 1 h, followed by incubation with Alexa 488- and Alexa 594-conjugated secondary antibodies and 4′,6-diamidino-2-phenylindole (DAPI) for 30 min in the dark. Coverslips were mounted onto glass slides using Perma Fluor and analyzed with confocal laser microscopy (Olympus FV3000 IX83). All procedures following the 2-hour incubation were performed at room temperature.

### *In vitro* replication assay

Parasites were added to MEF monolayers on coverslips at MOI = 1 and cultured for 48 h. Cells were then used for immunofluorescence staining, with GRA5 and GAP45 as PV and parasite markers, respectively. One hundred PVs per strain were counted for each experiment using confocal laser microscopy. Graphs represent means derived from three independent experiments. Two-way ANOVA was used to compare percentages of PVs containing the indicated numbers of parasites using GraphPad Prism 9.

### PV coating assay

MEFs were seeded at 1.0 × 10^5^ cells per well in a 6-well plate containing coated coverslips and treated with 10 ng/mL mouse IFN-γ for 18–20 hours at 37°C. Cells were infected with parasites at MOI = 4 and incubated at 37°C for 2 h.

PV coating was examined by immunofluorescence using primary antibodies against PV markers (GRA5 or GRA17) and host factors (Irgb6, Irga6, p62, GBP1, GBP2, and GBP1-5). Graphs represent means derived from four independent experiments. Student’s *t*-test was used to compare percentages of coated PVs using GraphPad Prism 9.

### Invasion assay

Parasite invasion was assessed in MEFs pre-treated with 10 ng/mL mouse IFN-γ using the method by Huynh et al. (44). “*Toxoplasma gondii* (1.B.516)” and anti-GAP45 antibodies were used to mark extracellular and intracellular parasites.

### Induced egress assay

MEFs were cultured overnight on coverslips with or without 10 ng/mL mouse IFN-γ. Freshly egressed parasites were added to MEFs and grown for 24–30 h at 37°C. The medium was replaced with serum-free DMEM containing 2 µM ionomycin (Nacalai Tesque) and incubated at 37°C for 5 min. Cells were fixed with 3.7% PFA and processed for immunofluorescence assay with anti-GAP45 and anti-GRA5 antibodies to stain parasites and PVs, respectively. At least 100 vacuoles were counted per strain and scored as occupied or egressed.

### Treatment of *T. gondii* with SNP and extracellular nitrite measurement

Parasites (2 × 10^6^/mL) were incubated at 37°C for 30 min in serum-free DMEM containing 0–2 mM SNP and then centrifuged (2,000 rpm for 5 min at 25°C). Supernatants were used for nitrite measurement with Griess Reagent Kit (Dojindo Laboratories). Cell fractions were used for MEF infection (MOI of 1) for luciferase assay.

#### *In vitro* measurement of *T. gondii* numbers by luciferase assay

MEFs were seeded at 1.0 × 10^5^ cells per well in a 12-well plate in DMEM containing 10% FCS ±10 ng/mL IFN-γ and incubated at 37°C for 18–20 h. Luc-expressing tachyzoites were added at an MOI of 1 and incubated at 37°C for 24 h.

BMDMs were seeded at 1.0 × 10^6^ cells/well in a 6-well plate in RPMI containing 5% L-cell supernatant and 10% FCS ± 10 ng/mL IFN-γ. Luc-expressing tachyzoites were added at an MOI of 0.05 and incubated at 37°C for 48 h.

Cells were collected and centrifuged (7,000 rpm for 5 min at 4°C) and suspended in 100 μL 1× Promega Passive Lysis Buffer, followed by sonication for 30 s. Cells were then centrifuged (14,000 rpm for 5 min at 4°C), and 5 μL of supernatants were mixed with 50 μL LARII and used for luciferase activity detection with a Promega GLOMAX20/20 luminometer. The data represent luciferase activity relative to that in untreated MEFs. Differences in the *T. gondii* inhibition activity between IFN-γ-activated vs non-activated or SNP-treated vs untreated conditions were analyzed by two-way ANOVA with Tukey’s multiple comparisons test to assess differences between genotypes.

### Ultrastructural expansion microscopy (U-ExM) of intracellular parasites

MEFs were cultured overnight on coverslips with or without 10 ng/mL mouse IFN-γ at 1.5 × 10^5^ cells per well in a 6-well plate. Cells were infected with *T. gondii* at MOI = 4 and incubated at 37°C for 2 h. Coverslips were washed once in PBS, then immersed in 1.4% formaldehyde/2% acrylamide (FA/AA) in PBS overnight at 37°C. From this step, the U-ExM protocol described by Fukumoto et al. (45).

### Bulk RNA-seq analysis

For RNA-seq analysis, total RNA was extracted from cells using an miRNeasy Mini kit (Qiagen) following the manufacturer’s instruction. Library preparation was performed using a TruSeq stranded mRNA sample prep kit (Illumina, San Diego, CA) according to the manufacturer’s instructions. Sequencing was performed on an Illumina NovaSeq 6000 sequencer (Illumina) in the 101-base single-end mode. Sequenced reads were mapped to the *Toxoplasma gondii* GT1 reference genome sequences (GCA_000149715.2_TGGT1) using HISAT2 version 2.1.0 (28), and the reads aligned to the *Toxoplasma* reference genome were counted using featureCounts (29). Raw count data were analyzed using iDEP.92 (30) and GSEA software ver. 4.1.0 (31). Genes with FDR < 0.05 and |log2FC| > 1 were considered as differentially expressed genes (DEGs). For GSEA, gene sets were obtained from published data. Heatmaps visualizing the normalized gene expression levels on a scale of rawmin to rawmax for each gene were generated using Morpheus (https://software.broadinstitute.org/morpheus).

### Mass spectrometry

Quantitative ubiquitylome analysis was performed based on the method described by Udeshi et al. (48), with several modifications as described below. WT and ΔTgJosephin parasites were cultured at 37℃ for 2 days in Vero cells, unstimulated MEFs, or MEFs stimulated overnight with 10 ng/mL IFN-γ. Parasites were filtered through a 5 μm filter and washed with HEPES-buffered saline. For each condition, approximately 1 × 10^8^ parasites were collected and lysed in 150 µL . Then, parasites were lysed in freshly prepared guanidine-TCEP buffer (6 M guanidine-HCl, 100 mM HEPES-NaOH, pH 7.5, 10 mM TCEP, 40 mM chloroacetamide) to avoid self-alkylation. Following heat denaturation at 95°C for 10 min and sonication, proteins were purified by methanol–chloroform precipitation and resuspended in 100 µL of 0.1% RapiGest SF (Waters) in 50 mM triethylammonium bicarbonate. After sonication and heating at 95°C for 10 min, proteins were digested with 2 µg of MS-grade trypsin (Thermo Fisher Scientific) at 37°C overnight. The resulting peptide solutions were heated again at 95°C for 10 min, diluted 6-fold with HBS (50 mM HEPES-NaOH, pH 7.5, 150 mM NaCl), and centrifuged. The supernatants were incubated with PTMScan HS K-ε-GG remnant magnetic immunoaffinity beads (Cell Signaling Technology) at 4°C for 2 h with rotation. The beads were used as supplied by the manufacturer, without additional antibody cross-linking, which differs from the protocol described by Udeshi et al. The beads were washed four times with HBS and resuspended in 100 µL of 100 mM HEPES-NaOH, pH 8.0. On-antibody tandem mass tag (TMT) labeling was performed by adding 0.25 mg of TMTpro 18-plex reagents (Thermo Fisher Scientific) and incubating at 25°C for 10 min with shaking. The reaction was quenched by adding 4 µL of 5% hydroxylamine. After two washes with Milli-Q water, the beads from all conditions were pooled, and peptides were eluted by incubating three times with 200 µL of 60% acetonitrile (ACN) and 0.1% trifluoroacetic acid (TFA). The combined eluates were evaporated, diluted to 120 µL with 10 mM ammonium formate (pH 9.0) in 2% ACN, and fractionated using offline high-pH reversed-phase chromatography on a Vanquish DUO UHPLC system (Thermo Fisher Scientific). Peptides were separated into 48 fractions, which were consolidated into 12 fractions. Each fraction was evaporated and dissolved in 3% ACN and 0.1% TFA. LC-MS/MS analysis of the resulting peptides was performed on an EASY-nLC 1200 UHPLC connected to a Q Exactive Plus mass spectrometer through a nanoelectrospray ion source (Thermo Fisher Scientific). The peptides were separated on a C18 reversed-phase column (75 μm × 150 mm; Nikkyo Technos) with a linear gradient of 4–32% ACN for 0–100 min, followed by an increase to 80% ACN for 10 min and a final hold at 80% ACN for 10 min. The mass spectrometer was operated in data-dependent acquisition mode with the top 10 MS/MS method. MS1 spectra were measured with a resolution of 70,000, an automatic gain control (AGC) target of 3e6, and a mass range of 375–1,400 *m*/*z*. HCD MS/MS spectra were acquired at a resolution of 35,000, an AGC target of 1e5, an isolation window of 0.7 *m*/*z*, a maximum injection time of 200 ms, and a normalized collision energy of 32. The dynamic exclusion was set to 20 s. Raw data were directly analyzed against *T. gondii* GT1 protein data (ToxoDB release 61) using Proteome Discoverer 2.5 (Thermo Fisher Scientific) with the Sequest HT search engine for identification and TMT quantification. The search parameters were as follows: (i) trypsin as an enzyme with up to two missed cleavages, (ii) precursor mass tolerance of 10 ppm, (iii) fragment mass tolerance of 0.02 Da, (iv) TMTpro of peptide N-terminus and carbamidomethylation of cysteine as fixed modifications, and (v) oxidation of methionine, TMTpro of lysine, and di-glycine of lysine as variable modifications. Peptides were filtered at a false discovery rate of 1% using the Percolator node.

## SUPPLEMENTAL MATERIAL

Figure S1 msphere.00137-26-s0001.tif Generation of single gene KO parasites.

Figure S2 msphere.00137-26-s0002.tif Mutations in mice.

Figure S3 msphere.00137-26-s0003.tif Homologues of TgRad23 and TgJosephin.

Figure S4 msphere.00137-26-s0004.tif SPM1 in parasites.

Figure S5 msphere.00137-26-s0005.tif *In vitro* growth and microtubule morphology of gene knockout parasites.

Legends msphere.00137-26-s0006.docx Legends for supplemental materials.

Table S1 msphere.00137-26-s0007.xlsx Primers, reagents, and tools used in this study.

Table S2 msphere.00137-26-s0008.xlsx Ubiquitin-targeted mass spectrometry in WT and ΔTgJosephin parasites.

Table S3 msphere.00137-26-s0009.xlsx Quantification of proteins in WT and ΔTgJosephin parasites by data-independent acquisition.

Table S4 msphere.00137-26-s0010.xlsx Bulk RNA sequencing in WT and ΔTgJosephin parasites.

ASM does not own the copyrights to Supplemental Material that may be linked to, or accessed through, an article. The authors have granted ASM a non-exclusive, world-wide license to publish the Supplemental Material files. Please contact the corresponding author directly for reuse.
