# Supplementary figures and images for: TgJosephin and TgRad23 are important for anti-IFN-γ virulence via deubiquitination of SPM1 in Toxoplasma
Source: mSphere. 2026 Apr 13;11(5):e00137-26. doi: 10.1128/msphere.00137-26 (PMC13203982; doi:10.1128/msphere.00137-26)

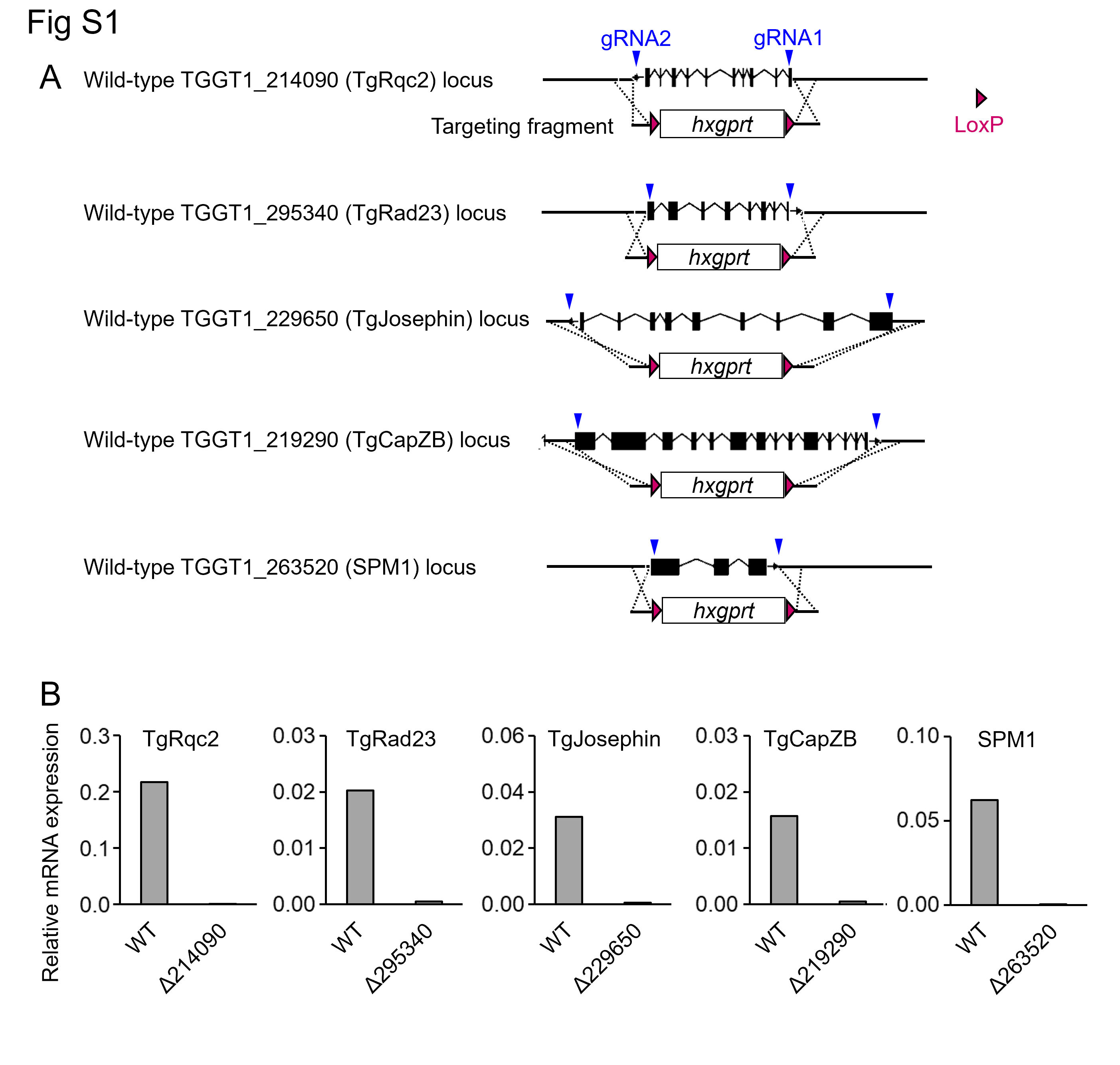

Supplement: Figure S1 — Generation of single gene KO parasites. [file msphere.00137-26-s0001.tif]

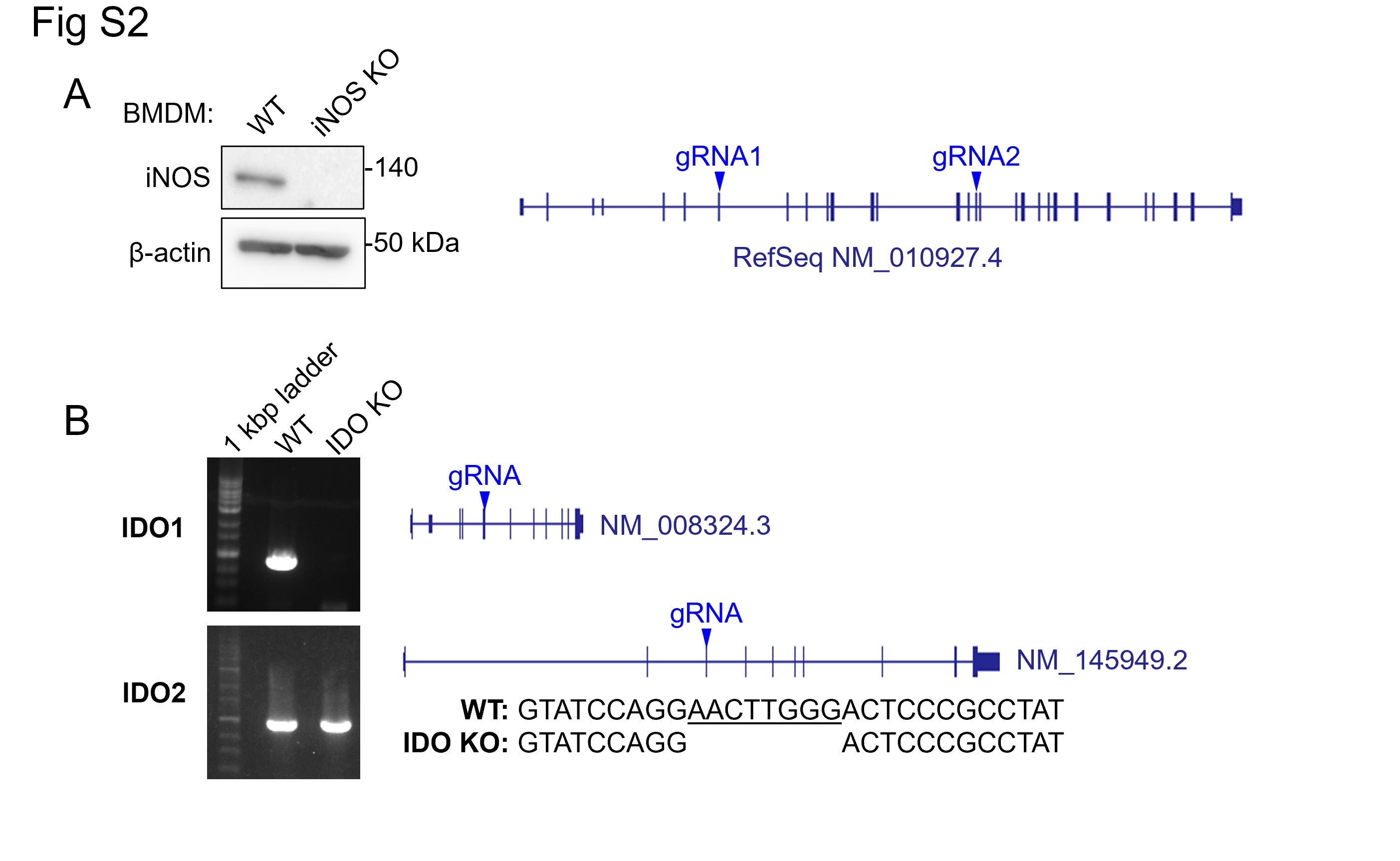

Supplement: Figure S2 — Mutations in mice. [file msphere.00137-26-s0002.tif]

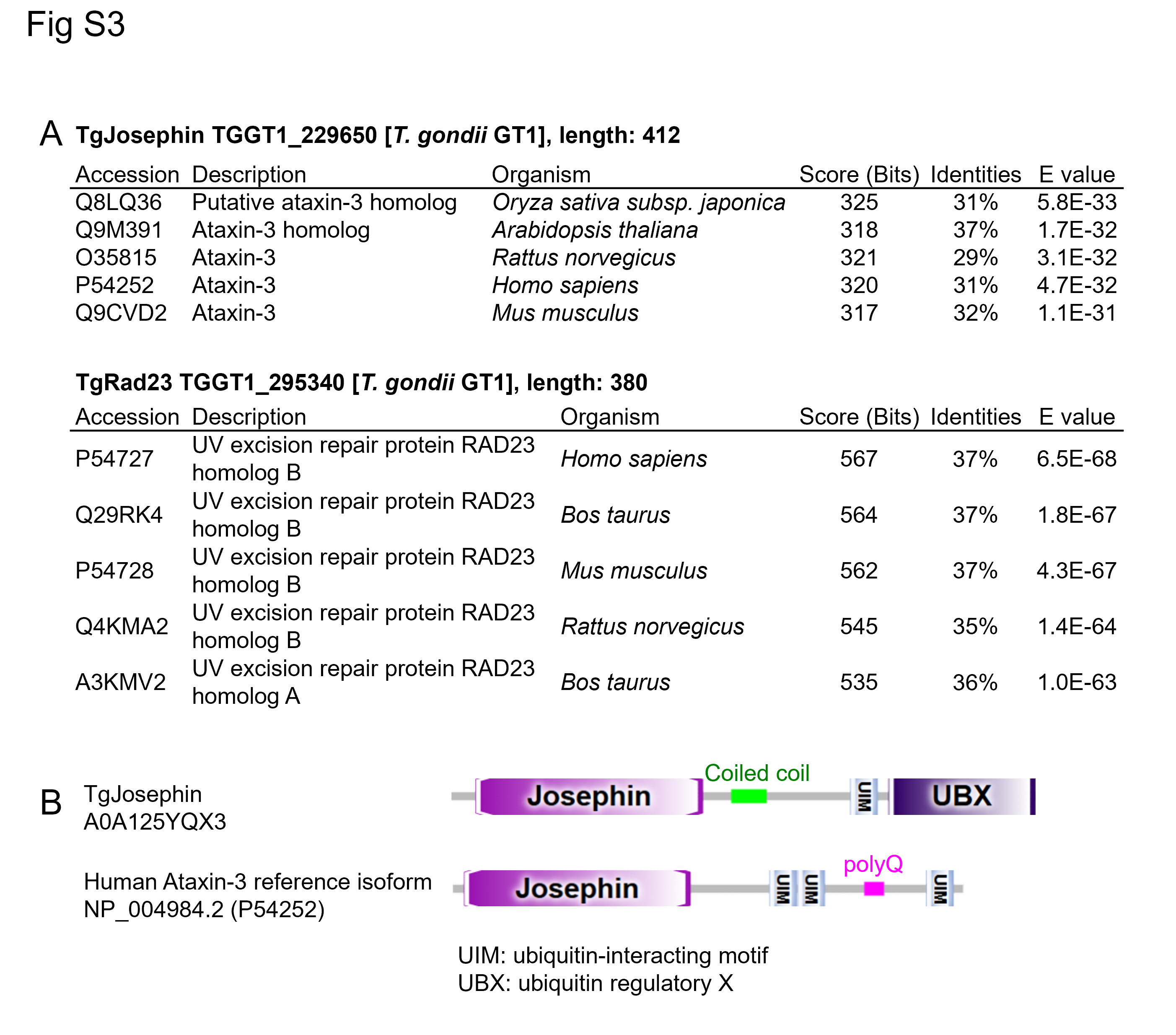

Supplement: Figure S3 — Homologues of TgRad23 and TgJosephin. [file msphere.00137-26-s0003.tif]

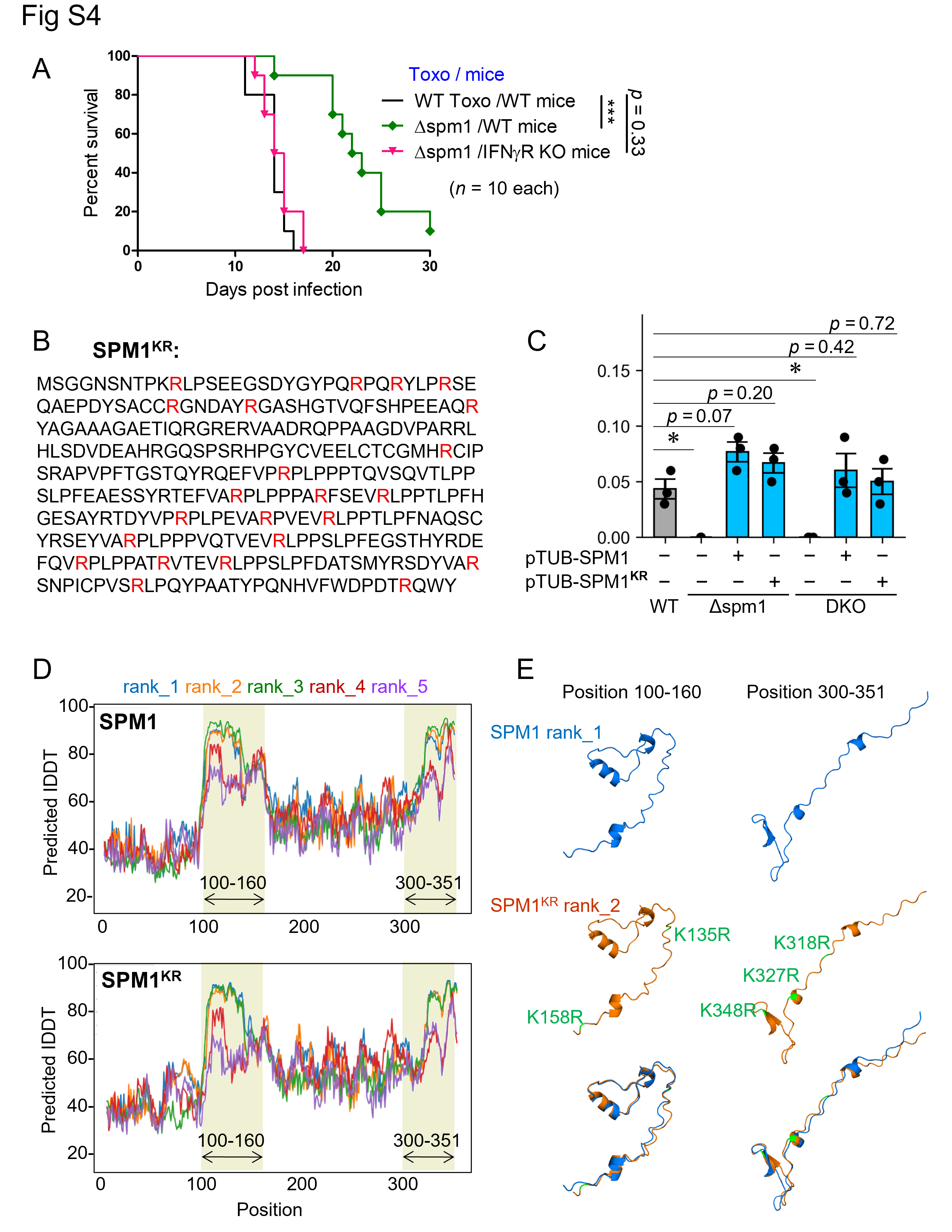

Supplement: Figure S4 — SPM1 in parasites. [file msphere.00137-26-s0004.tif]

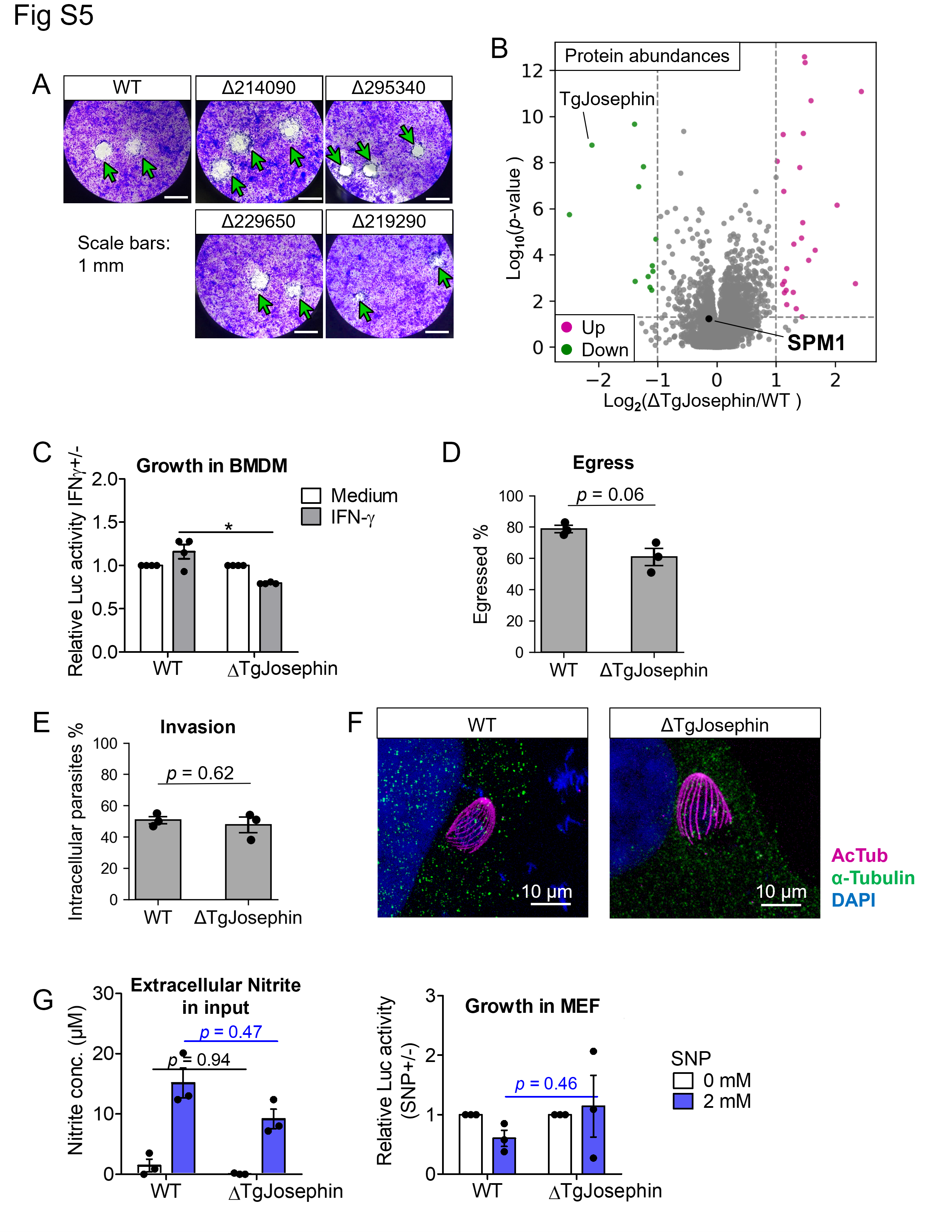

Supplement: Figure S5 — In vitro growth and microtubule morphology of gene knockout parasites. [file msphere.00137-26-s0005.tif]
