# Supplementary material for: TgJosephin and TgRad23 are important for anti-IFN-γ virulence via deubiquitination of SPM1 in Toxoplasma
Source: mSphere. 2026 Apr 13;11(5):e00137-26. doi: 10.1128/msphere.00137-26 (PMC13203982; doi:10.1128/msphere.00137-26)
Supplement: Legends — for supplemental materials. [file msphere.00137-26-s0006.docx]

## **Supplementary Figure S1. Generation of single gene KO parasites.**

1. Deletion of genes. The WT locus of each gene was cleaved by CRISPR/Cas9 at the indicated gRNA target sites (1), and a PCR fragment containing the HXGPRT cDNA with two flanking LoxP sequences was inserted.
2. RT-qPCR analysis of parasites. Expression levels of the indicated genes in parental (WT) and mutant parasites were normalized to TgACT1 mRNA.

## **Supplementary Figure S2. Mutations in mice.**

1. iNOS expression in mice: western blotting analysis of 1μg/mL LPS-stimulated BMDMs induced from WT and iNOS KO mice (left). The gRNA target site on the iNOS gene is shown (right) (2, 3).
2. Gel electrophoresis images of PCR products made from WT and IDO-deficient mouse tail fibroblast genome templates (left). 0.9-1.0 kbp exon regions including the CRISPR/Cas9 target sites in IDO1 and IDO2 were amplified (right). The underlined sequence at the IDO2 cleavage site was absent in IDO KO samples, causing a codon frame shift (2, 3).

## **Supplementary Figure S3. Homologues of TgRad23 and TgJosephin.**

1. The top five hits for *T. gondii* TgJosephin and TgRad23 full protein sequences by PSIBLAST 2.14.0+. Database: uniprotkb_swissprot (4).
2. Protein domains of TgJosephin and human Ataxin-3 predicted in SMART (5).

## **Supplementary Figure S4. SPM1 in parasites.**

1. Survival analyses of WT or IFNγR-deficient mice infected with WT or SPM1-deficient parasites.
2. Amino acid sequence of SPM1^KR^. Lysine-to-arginine substitutions are highlighted in red.
3. RT-qPCR analysis of WT, Δspm1, and Δspm1ΔTgJosephin DKO parasites. mRNA levels of SPM1/SPM1^KR^ expressed by the endogenous promoter (grey) or the TUBA1 promoter (blue) were normalized to TgACT1 mRNA. Graph represents mean ± sem, *n* = 3.
4. IDDT (Local Distance Difference Test) scores of SPM1 and SPM1^KR^ rank_1 to rank_5, predicted with ColabFold(6).
5. Unrelaxed Alphafold2 models of positions 100-160 and 300-351 of SPM1 rank_1 and SPM1^KR^ rank_2, created with ColabFold and aligned using PyMOL(7).

**p* < 0.05; ****p* < 0.001.

## **Supplementary Figure S5. *In vitro* growth and microtubule morphology of gene knockout parasites.**

1. Optical microscope images of plaques made by *T. gondii* in MEFs. Plaques are indicated with arrowheads.
2. Volcano plot of *T. gondii* proteins in WT and ΔTgJosephin parasites grown in Vero cell dishes, quantified by data-independent acquisition (DIA). Each dot represents a *T. gondii* peptide. *n* = 5.
3. Relative luciferase counts of parasites grown for 48 hours in BMDMs (MOI = 0.05) with or without 10 ng/mL IFN-γ pre-treatment. Data shown are luciferase count ratios of IFN-γ-treated/untreated samples for each parasite line, *n* = 4.
4. Ionophore-induced egress assay of parasites in MEF with 10 ng/mL IFN-γ treatment, *n* = 3.
5. Invasion assay of parasites in MEFs with 10 ng/mL IFN-γ pre-treatment, *n* = 3.
6. Parasite microtubules in 10 ng/mL IFNγ-treated MEFs, visualized with ultrastructural expansion microscopy (U-ExM). Confocal microscope images were taken with a 100x lens.
7. Effect of extracellular nitric oxide on parasites *in vitro*, *n* = 3. Left: nitrite measurement of parasite supernatants (2×10^6^ parasites/mL) after incubation in serum-free DMEM containing 2 mM SNP for 30 min. Right: relative luciferase counts of parasites grown in MEFs (MOI = 1) for 24 hours after exposure to 2 mM SNP. Data shown are luciferase count ratios of 2 mM SNP-treated/untreated (0 mM) samples for each parasite line.

Bar graphs represent mean ± sem.

## **Supplementary Table S1. Primers, reagents, and tools used in this study.**

1. Primers used in this study. UPRT_gRNA_F and UPRT_gRNA_R were described previously (8).
2. Reagents and tools used in this study. The anti-GRA17 antibody was described previously (9).

## **Supplementary Table S2. Ubiquitin-targeted mass spectrometry in WT and ΔTgJosephin parasites.**

1. Data from parasites in Vero cells, *n* = 5.
2. Data from parasites in MEFs unstimulated (*n* = 4) or stimulated with IFN-γ (*n* = 5).

## **Supplementary Table S3. Quantification of proteins in WT and ΔTgJosephin parasites by data-independent acquisition (DIA).**

Quantification of *T. gondii* proteins in WT and ΔTgJosephin parasites in Vero cells, *n* = 5.

## **Supplementary Table S4. Bulk RNA sequencing in WT and ΔTgJosephin parasites.**

Quantification of *T. gondii* RNA sequences detected in WT and ΔTgJosephin parasites in Vero cells, *n* = 3.

1. Gajria B, Bahl A, Brestelli J, Dommer J, Fischer S, Gao X, Heiges M, Iodice J, Kissinger JC, Mackey AJ, Pinney DF, Roos DS, Stoeckert CJ Jr, Wang H, Brunk BP. 2008. ToxoDB: an integrated Toxoplasma gondii database resource. Nucleic Acids Res 36:D553–D556.

2. Perez G, Barber GP, Benet-Pages A, Casper J, Clawson H, Diekhans M, Fischer C, Gonzalez JN, Hinrichs AS, Lee CM, Nassar LR, Raney BJ, Speir ML, van Baren MJ, Vaske CJ, Haussler D, Kent WJ, Haeussler M. 2024. The UCSC Genome Browser database: 2025 update. Nucleic Acids Res 53:D1243–D1249.

3. O’Leary NA, Cox E, Holmes JB, Anderson WR, Falk R, Hem V, Tsuchiya MTN, Schuler GD, Zhang X, Torcivia J, Ketter A, Breen L, Cothran J, Bajwa H, Tinne J, Meric PA, Hlavina W, Schneider VA. 2024. Exploring and retrieving sequence and metadata for species across the tree of life with NCBI Datasets. Sci Data 11:732.

4. Madeira F, Madhusoodanan N, Lee J, Eusebi A, Niewielska A, Tivey ARN, Lopez R, Butcher S. 2024. The EMBL-EBI Job Dispatcher sequence analysis tools framework in 2024. Nucleic Acids Res 52:W521–W525.

5. Schultz J, Copley Rr, Doerks T, Ponting Cp, Bork P. 2000. SMART: a web-based tool for the study of genetically mobile domains. Nucleic Acids Res 28.

6. Mirdita M, Schütze K, Moriwaki Y, Heo L, Ovchinnikov S, Steinegger M. 2022. ColabFold: Making Protein folding accessible to all. Nat Methods https://doi.org/10.1038/s41592-022-01488-1.

7. The PyMOL Molecular Graphics System (Version 3.0). Schrödinger, LLC.

8. Tachibana Yuta, Sasai Miwa, Yamamoto Masahiro. 2024. CRISPR screens identify genes essential for in vivo virulence among proteins of hyperLOPIT-unassigned subcellular localization in Toxoplasma. mBio 15:e01728-24.

9. Tachibana Y, Hashizaki E, Sasai M, Yamamoto M. 2023. Host genetics highlights IFN-γ-dependent *Toxoplasma* genes encoding secreted and non-secreted virulence factors in *in vivo* CRISPR screens. Cell Rep 42:112592.
